# Clinical Significance of an m6A Reader Gene, *IGF2BP2*, in Head and Neck Squamous Cell Carcinoma

**DOI:** 10.3389/fmolb.2020.00068

**Published:** 2020-04-24

**Authors:** Xiaoli Deng, Qingshan Jiang, Zhifeng Liu, Wen Chen

**Affiliations:** ^1^Key Laboratory of Xinjiang Phytomedicine Resource and Utilization, Ministry of Education, School of Pharmacy, Shihezi University, Shihezi, China; ^2^Department of Otorhinolaryngology, The First Affiliated Hospital of University of South China, Hengyang, China

**Keywords:** head and neck squamous cell carcinoma (HNSCC), insulin-like growth factor 2 mRNA-binding protein 2 (IGF2BP2), N6-methyladenosine (m6A), prognostic factor, tumor

## Abstract

The importance of N6-methyladenosine (m6A) in tumor recurrence and prognosis has been recognized in recent years. The role of m6A readers, such as insulin-like growth factor 2 mRNA-binding protein 2 (IGF2BP2), in regulating head and neck squamous cell carcinoma (HNSCC) remains unclear. We, therefore, assessed the prognostic role of IGF2BP2 in HNSCC using openly available data from The Cancer Genome Atlas (TCGA) in conjunction with HNSCC patient sample immunohistochemistry (*n* = 36). The correlation between *IGF2BP2* expression and clinical characteristics was then examined. The role of IGF2BP2 in prognosis was assessed by Kaplan–Meier curves and Cox analysis. Finally, TCGA data was used to explore possible carcinogenic mechanisms with multi-GSEA (gene set enrichment analysis). Analysis of TCGA data and IHC results revealed that *IGF2BP2* was upregulated in HNSCC tumor tissues, and the expression level was related to the T stage. Simultaneously, Kaplan–Meier curves and Cox analysis indicated that highly expressed *IGF2BP2* correlated with poor prognosis and that *IGF2BP2* was a potential prognostic factor for HNSCC. Gene set enrichment analysis revealed that scavenging and degradation, synthesis and metabolism, cell growth, death and motility, and cancer pathways were differentially enriched in patients with high *IGF2BP2* expression. Our results demonstrate that *IGF2BP2* plays an important role in tumor progression and may serve as an important biological prognostic factor for HNSCC.

## Introduction

The global incidence of head and neck cancers is increasing (Global Burden of Disease Cancer et al., 2015). Head and neck squamous cell carcinoma (HNSCC) accounts for more than 90% of these malignancies and is usually related to smoking and drinking habits. Nevertheless, a few young people with no alcohol and smoking history may develop HNSCC due to HPV infection (Cramer et al., 2019). Despite continuous innovations in HNSCC treatment methods such as surgery, chemoradiotherapy, and immunotherapy, its 5-year survival rate has not improved significantly (Siegel et al., 2018). Therefore, it is of great importance to find specific molecular markers of HNSCC to better understand its progression and develop novel therapies.

N6-methyladenosine (m6A) is the most common modification of eukaryotic mRNA, characterized by methylation of the N6-position of adenosine. The role of m6A in cancer has just begun to be recognized (Deng et al., 2018). Studies have found that methyltransferases (writers), demethylases (erasers), and binding proteins (readers) regulate m6A (Panneerdoss et al., 2018), while insulin-like growth factor 2 mRNA-binding protein 2 (IGF2BP2) acts as a reader (Hu et al., 2019). IGF2BP2 is also an RNA-binding protein (RBP) that serves as a post-transcriptional regulatory factor for mRNA localization, stability, and translational control (Huang et al., 2018). IGF2BP2 imbalance can lead to the occurrence of many diseases, including insulin resistance, obesity, and cancer. At present, IGF2BP2 has been identified as a novel stage-specific biomarker of colorectal cancer progression. However, whether IGF2BP2 could also be a specific marker in HNSCC is yet to be determined.

In the present study, we have evaluated the expression of *IGF2BP2* in HNSCC. We have analyzed the relationship between *IGF2BP2* expression and clinical features, as well as explored the potential prognostic significance of IGF2BP2 in HNSCC patients. Multi-gene set enrichment analysis (GSEA) was performed to gain further insight into the biological pathways involved in HNSCC pathogenesis related to the IGF2BP2 regulatory mechanism.

## Materials and Methods

### Data Mining and Collection

The TCGA HNSC data (528 cases, Workflow Type: HTSeq-Counts) was downloaded from the GDC Data Portal of the National Cancer Institute^1^. The dataset contains survival data with clinical information and mRNA expression counts. The samples with missing expression data were excluded from the study. The RNA-Seq gene expression level 3 HTSeq-Counts data of 501 patients with HNSCC and clinical data were retained and further analyzed. According to the database guidelines, the datasets may be used for publication without restriction or limitation.

### Data Analysis

The acquired data were analyzed using R (v.3.4.3). Logistic regression and the KS test were used to analyze the correlation between the expression level of the *IGF2BP2* gene and clinicopathological features. Cox regression and the Kaplan-Meier curve were used to analyze the overall survival of HNSCC patients with different clinicopathological parameters from the data in TCGA. Finally, we compared the correlation between the expression level of *IGF2BP2* and the clinical parameters [age (years ≥60/<60), gender (male/female), grade (G1/G2/G3/G4/Gx), stage (I/II/III/IV), local invasion (T1/2/3/4/Tx), lymph node involvement (positive/negative), distant metastasis (M0/M1/Mx), and HPV infection (positive/negative)] related to survival using the multivariate Cox analysis of influencing factors. The cut-off value of *IGF2BP2* expression was determined by Cutoff Finder.^2^

### Gene Set Enrichment Analysis

GSEA is a computational method used to determine statistical differences between two biological expression states in *a priori* defined set of genes (Subramanian et al., 2005). In this study, GSEA generated an ordered list of genes based on the pathways which were related to the expression level of *IGF2BP2* and then annotated the significant differences between the high- and low-level expression groups of *IGF2BP2*. Each gene set was calculated 1,000 times in each analysis. The expression level of the *IGF2BP2* gene acts as a phenotypic label. The signaling pathway enrichment analysis of the phenotypes and the results of the multi-GSEA were ranked by their nominal *p*-value and normalized enrichment score (NES).

### Tissue Preparation and Immunohistochemistry

In total, 36 sets of pathological sections of HNSCC were provided by the First Affiliated Hospital of the University of South China. The sections were placed in 10 mM citrate buffer solution (pH = 6.0) and microwaved for 15 min for antigen retrieval. After restoration to 37°C, 3% hydrogen peroxide was added and incubated for 10 min at 37°C to deplete the activity of endogenous peroxidase. The sections were incubated with an anti-IGF2BP2 polyclonal antibody (Catalog number: 11601-1-AP, Proteintech, Wuhan, China) at 4°C for 24 h, with the optimal dilution ratio of 1:100. After rinsing thoroughly with phosphate buffer solution (PBS), the sections were immunohistochemically colored with horseradish peroxidase-labeled goat anti-rabbit polymers. Finally, the positive expression of the IGF2BP2 protein was observed with the incubated chromogenic agent, 3’,3’-diaminobenzidine, and the nuclei were counter-stained with Mayer’s hematoxylin. The immunohistochemical staining results of the sections were scored by two different experimenters, blindly. When the experimenters scored sections differently, the sections were re-examined by a third experimenter to reach an agreement. Signal strength was scored in the following manner: 0 (no signal), 1 (weak), 2 (strong). The staining distribution score is based on the percentage of positive cells: 0 (0%), 1 (1–50%), and 2 (51–100%).

## Results

### Clinicopathological Features of the Patients

**FIGURE 1:**
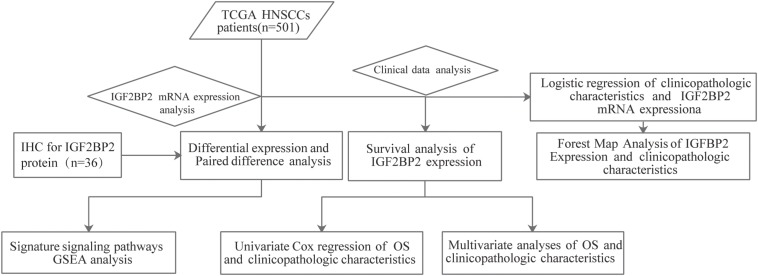
Workflow graph for this study.

The workflow graph of this study is summarized in Figure 1. The gene expression and clinical data of the 501 patients were downloaded from The Cancer Genome Atlas (TCGA) database. The detailed clinical features, including TNM staging, HPV infection status, and survival status, are shown in Table 1.

**TABLE 1:** Clinical characteristics of TCGA HNSCCs patients (*n* = 501).

Clinical characteristics	Total (501)	%
**Age**		
<60	221	44.1
≥60	279	55.7
NA	1	0.2
**Gender**		
Female	134	26.7
Male	367	73.3
**Histologic grade**		
G1–2	362	72.3
G3–4	120	24.0
Gx	16	3.2
NA	3	0.6
**Stage**		
I–II	95	19.0
III–IV	338	67.5
NA	68	13.6
**T classification**		
T1–2	178	35.5
T3–4	267	53.3
Tx	34	6.8
NA	22	4.4
**N classification**		
N0	170	33.9
N1–3	238	47.5
Nx	69	13.8
NA	24	4.8
**M classification**		
M0	187	37.3
M1	1	0.2
Mx	61	12.2
NA	252	50.3
**Vital status**		
Deceased	196	39.1
Living	305	60.9
**HPV infection**		
Positive	31	6.2
Negative	72	14.4
NA	398	79.4

### High *IGF2BP2* mRNA Expression in HNSCC

**FIGURE 2:**
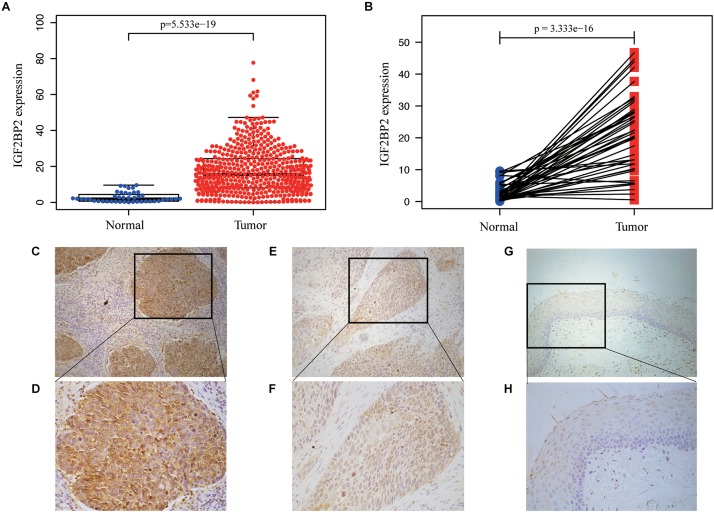
IGF2BP2 is overexpressed in HNSCC. **(A)** 354 high IGF2BP2 mRNA expression in HNSCC based on TCGA DATA. **(B)** Paired difference analysis of IGF2BP2 mRNA expression in HNSCC based on TCGA DATA. **(C,D)** Representative immunohistochemical staining for IGF2BP2 protein in 36 matched HNSCC tissues and corresponding adjacent non-cancerous epithelial tissues. High expression of IGF2BP2 protein in primary HNSCC specimens (200x, 400x). **(E,F)** Low expression of IGF2BP2 protein in HNSCC specimens (200x, 400x). **(G,H)** Negative expression of IGF2BP2 in adjacent non-cancerous epithelial tissues (200x, 400x).

The TCGA database provides a unique opportunity to understand the role of *IGF2BP2* in HNSCC. We analyzed the differences in the expression of *IGF2BP2* between HNSCC tumor tissues and adjacent tissues through differential expression scatter plots and paired difference analyses. As shown in Figure 2A, the expression level of *IGF2BP2* was, statistically, higher in HNSCC tumor tissues (*p* = 5.533e-19) compared to adjacent tissues. As shown in Figure 2B, the expression of *IGF2BP2* in paired cancer tissues was also highly statistically significant (*p* = 3.333e-16). Then, we verified the level of *IGF2BP2* protein expression in 36 pairs of HNSCC tumor tissues and adjacent tissues by immunohistochemistry staining and found significant elevated *IGF2BP2* expression in terms of density and intensity in HNSCC tumor tissues compared with adjacent tissues (Figures 2C–H).

### Correlation Between Clinicopathological Features and *IGF2BP2* Expression in HNSCC

The correlation between the clinicopathological features and the expression of *IGF2BP2* is shown in Table 2. The expression of *IGF2BP2* was highly statistically significant correlated with local invasion (T1–2 vs. 3–4, *p* = 0.023) and HPV infection (positive vs. negative, *p* = 0.001).

**TABLE 2:** Correlation between the clinicopathologic characteristics and IGF2BP2 mRNA expression^*a*^ (logistic regression).

Clinical characteristics	Total (N)	Odds ratio in IGF2BP2 expression	*p*-Value
Age (≥60 vs. <60)	501	0.935 (0.657–1.331)	0.711
Grade (G1–2 vs. G3–4)	482	1.007 (0.990–1.026)	0.41
Stage (I–II vs. III–IV)	284	1.015 (0.994–1.036)	0.169
Local invasion (T1–2 vs. 3–4)	445	1.017 (1.002–1.032)	**0.023**
Lymph nodes (positive vs. negative)	408	1.009 (0.989–1.030)	0.369
HPV infection (positive vs. negative)	103	4.800 (1.831–12.580)	**0.001**

*^*a*^Categorical dependent variable, greater or less than the cutoff expression level. Bold values indicate statistically significant, P < 0.05.*

### Correlation Between *IGF2BP2* Expression and Survival

**FIGURE 3:**
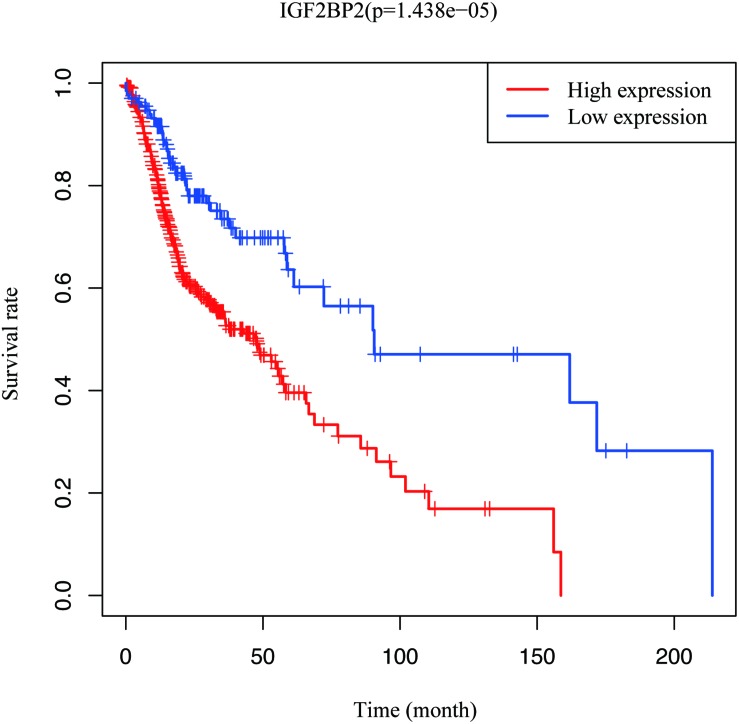
Survival analysis of IGF2BP2 expression in terms of overall survival. Kaplan–Meier curves produced survival analysis.

**FIGURE 4:**
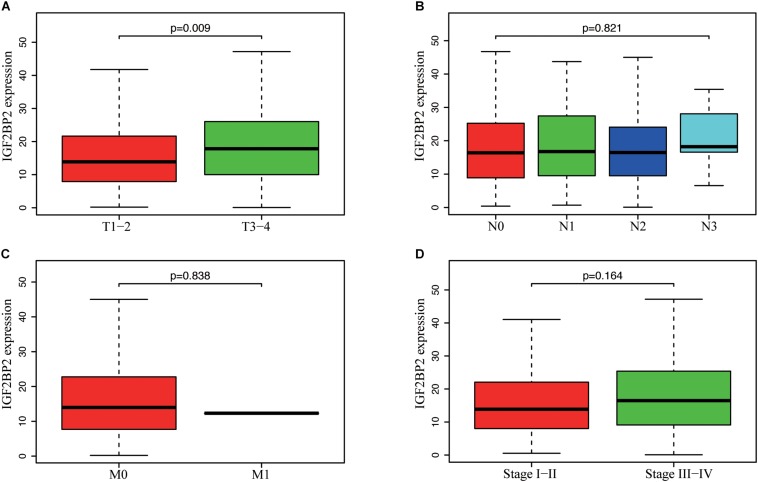
Correlation between IGF2BP2 expression and clinicopathologic characteristics. **(A)** subgroup analysis of T classification (T1–2 and T3–T4). **(B)** Subgroup analysis of N classification (N0/N1/N2/N3). **(C)** Subgroup analysis of M classification (M0 and M1). **(D)** Subgroup analysis of clinical stage (stage I–II and stage III–IV). Wilcox test in A/C/D, Kruskal test in **(B)**.

**FIGURE 5:**
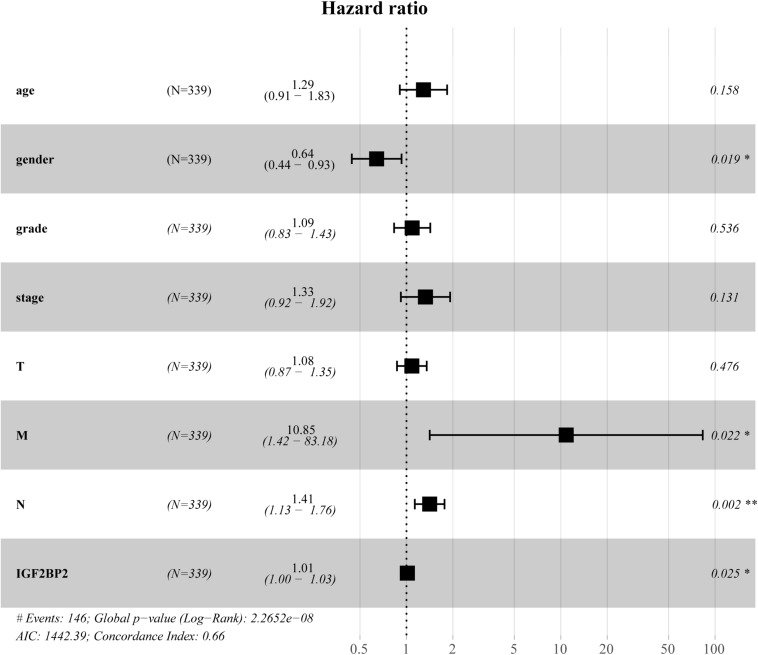
Forest Map Analysis of Expression and clinicopathologic characteristics.

To estimate the effect of *IGF2BP2* on the prognosis of HNSCC patients, we used the Kaplan-Meier survival analysis and log-rank test to evaluate the correlation between *IGF2BP2* expression and overall survival. The survival of patients with high *IGF2BP2* expression was relatively poor (*p* = 1.438e-5; Figure 3). The subgroup analysis indicated that the patients in the T1–2 group vs. the T3–4 group with a high expression level of IGF2BP2 also had statistically significant poor overall survival (*p* = 0.009; Figure 4A) but not in the N classification, M classification or clinical stage (Figures 4B–D). Univariate analysis was performed with the variables listed in Table 3. Multivariate analysis with the Cox proportional hazards model indicated that the expression of *IGF2BP2* (*HR* = 1.013, *p* = 0.028) was a potential prognostic factor for patients with HNSCC (Table 4). Through the forest plot analysis (Figure 5), there are statistically significant linkages between gender (*p* = 0.019), distant metastasis (*p* = 0.022), neck lymph node metastasis (*p* = 0.002), the expression of *IGF2BP2* (*p* = 0.025) and the outcome of HNSCC patients. In general, IGF2BP2 may serve as a reliable and effective independent prognostic biomarker.

**TABLE 3:** Univariate cox regression of overall survival and clinicopathologic characteristics in TCGA HNSCCs patients.

Clinical characteristics (Univariate cox regression)	Hazard ratio	HR (95% CI)	*p*-value
Age, years (≥60/<60)	1.301	0.974–1.738	0.075
Gender (male/female)	1.366	1.013–1.841	**0.041**
IGF2BP2 expression (low/high)	1.014	1.004–1.025	**0.007**
Grade (G1/G2/G3/G4/Gx)	1.061	0.898–1.254	0.487
Stage (I/II/III/IV)	1.382	1.148–1.663	**0.001**
Local invasion (T1/2/3/4/Tx)	1.153	1.028–1.294	**0.015**
Lymph nodes (positive/negative)	1.164	1.071–1.264	**0.000**
Distant metastasis (M0/M1/Mx)	0.974	0.751–1.320	0.974
HPV infection (positive/negative)	1.808	0.519–6.293	0.352

*Bold values indicate statistically significant, P < 0.05.*

**TABLE 4:** Multivariate analyses of overall survival and clinicopathologic characteristics in TCGA HNSCCs patients.

Clinical characteristics (multivariate analyses)	Hazard ratio	HR (95% CI)	*p*-Value
Gender (male/female)	1.454	1.046–2.020	**0.026**
IGF2BP2 expression (low/high)	1.013	1.001–1.026	**0.028**
Stage (I/II/III/IV)	1.032	0.726–1.468	0.861
Local invasion (T1/2/3/4/Tx)	1.183	0.916–1.528	0.197
Lymph nodes (positive/negative)	1.147	1.025–1.284	**0.017**

*Bold values indicate statistically significant, P < 0.05.*

### GSEA Identifies an *IGF2BP2*-Related Signaling Pathway

**FIGURE 6:**
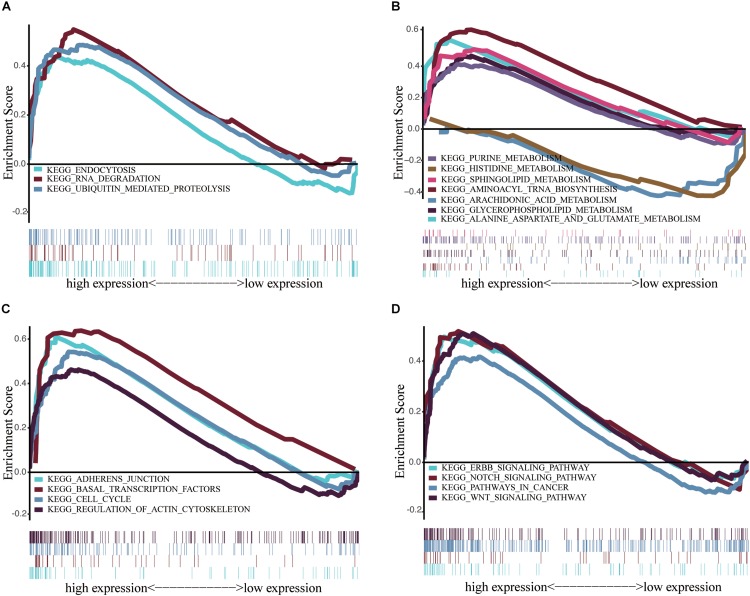
Enrichment plots from multiple GSEA. **(A)** Scavenging and degradation: Endocytosis, RNA degradation and ubiquitin mediated proteolysis. **(B)** Synthesis and metabolism: Alanine aspartate and glutamate metabolism, aminoacyl tRNA biosynthesis, arachidonic acid metabolism glycerophospholipid metabolism, histidine metabolism, purine metabolism, sphingolipid metabolism. **(C)** Cell growth, death, and motility: adherens junction, basal transcription factors, cell cycle, regulation of actin cytoskeletion. **(D)** Cancer pathways and Others: ERBB signaling pathway, Notch signaling pathway, pathways in cancer, Wnt signaling pathway.

We performed GSEA to identify differentially regulated pathways between groups with high and low expression of *IGF2BP2* and determine the activated signaling pathways in HNSCC. We selected results with significant differences in enrichment (FDR < 0.25, NOM *p* < 0.05) in the MSigDB gene set (c2.cp.kegg.v6.2.symbols.gmt) (Table 5). The most significantly enriched signaling pathways were selected based on the NES. Figure 6 shows that biological clearance and degradation, synthesis and metabolism, cell growth, death and motility, and cancer pathways were significantly enriched in the group with high expression levels of *IGF2BP2*.

**TABLE 5:** Gene sets enriched in phenotype high and low.

MSigDB collection	Gene set name	NES	NOM *p*-val	FDR *q*-val
c2.cp.kegg. v6.2.symbols.gmt	KEGG_ENDOCYTOSIS	1.718	0.010	0.145
	KEGG_RNA_DEGRADATION	1.806	0.012	0.173
	KEGG_UBIQUITIN_MEDIATED_PROTEOLYSIS	1.711	0.029	0.142
	KEGG_ALANINE_ASPARTATE_AND_GLUTAMATE_METABOLISM	1.766	0.006	0.171
	KEGG_AMINOACYL_TRNA_BIOSYNTHESIS	1.821	0.016	0.186
	KEGG_ARACHIDONIC_ACID_METABOLISM	1.531	0.021	0.674
	KEGG_GLYCEROPHOSPHOLIPID_METABOLISM	1.742	0.006	0.161
	KEGG_HISTIDINE_METABOLISM	1.473	0.045	0.626
	KEGG_PURINE_METABOLISM	1.576	0.028	0.166
	KEGG_SPHINGOLIPID_METABOLISM	1.670	0.024	0.129
	KEGG_ADHERENS_JUNCTION	1.890	0.002	0.191
	KEGG_BASAL_TRANSCRIPTION_FACTORS	1.874	0.004	0.150
	KEGG_CELL_CYCLE	1.744	0.033	0.178
	KEGG_REGULATION_OF_ACTIN_CYTOSKELETON	1.729	0.010	0.160
	KEGG_PATHWAYS_IN_CANCER	1.622	0.029	0.138
	KEGG_WNT_SIGNALING_PATHWAY	1.937	0.000	0.204
	KEGG_ERBB_SIGNALING_PATHWAY	1.710	0.013	0.132
	KEGG_NOTCH_SIGNALING_PATHWAY	1.678	0.029	0.138

*Gene sets with NOM P < 0.05 and FDR q < 0.25 are considered as significant. FDR, false discovery rate; NES, normalized enrichment score; NOM, nominal.*

## Discussion

This study illustrated the importance of *IGF2BP2*, a potential biomarker for the prognosis of HNSCC patients. We found a high expression of *IGF2BP2* in HNSCC tumor tissues and verified this result in our laboratory using immunohistochemistry. This is consistent with previous studies of *IGF2BP2* in cancers, including colorectal cancer (Wang et al., 2019), gastrointestinal cancer (Zhou et al., 2017), endometrial cancer (Hiramatsu et al., 2016), pancreas cancer (Xu et al., 2019), breast cancer (McMullen et al., 2018), and hepatic carcinoma. We found that the upregulation of *IGF2BP2* had some statistical correlation with the T stage, HPV status, and overall survival in HNSCC. These results suggest that targeting *IGF2BP2* may provide a novel treatment for HNSCC, especially in cases that are HPV-driven. Most importantly, we inferred that I*GF2BP2* expression is an independent prognostic predictor for the survival of HNSCC and may be a promising biomarker for clinical use. Although our analysis of *IGF2BP2* expression does not effectively predict disease-free survival (data not shown), it is closely related to the locally advanced neck and lymph node metastasis in HNSCC patients.

Most of the previous research on *IGF2BP2* has focused on its role as a retinol-binding protein (RBP) targeting downstream transcription factors to regulate post-transcriptional translational carcinogenesis. In cervical cancer, gastric carcinoma proliferation enhancing transcript 1 (GHET1) regulates AKT/mTOR and Wnt/β-catenin signaling pathways by stabilizing the interaction of E2F6 and IGF2BP2 (Liu et al., 2020). Non-coding genes can also target IGF2BP2 directly: the lncRNA HOTAIR inhibits IGF2BP2 and regulates colon cancer growth and invasion (Wu et al., 2018). IGF2BP2 activity can be mediated by mTOR, a major effector downstream of PI3K/AKT signaling (Lai et al., 2016). Additionally, miR-141 directly targets IGF2BP2 and can reduce PI3K/AKT activity and inhibit pancreatic cancer proliferation (Xu et al., 2019).

In recent years, IGF2BP2 has been found to act as a reader of m6a to regulate carcinogenesis progression. Studies in colon carcinoma have shown that METTL3 stabilizes the expression of SOX2 by an m6A-IGF2BP2-dependent mechanism (Li et al., 2019). Decreased m6A methylation levels are important in endometrial cancer, which is highly associated with the AKT signaling pathway (Liu et al., 2018).

IGF2BP2 expression helps maintain tumor stemness (Kessler et al., 2015). P62, an alternate form of IGF2BP2 through alternative splicing, is highly expressed in hepatocellular carcinoma, which presents with the characteristics of stemness and high blood vessel distribution. The stem gene DLK1 is overexpressed in mice that overexpress P62, and tumor formation is more aggressive due to cancer stemness (Kessler et al., 2015). This regulation of tumor stemness may also be related to chemotherapy resistance and lead to tumor recurrence. The downregulation of *IGF2BP2* can induce chemosensitivity, similar to the inhibition of the PI3K/AKT signaling pathway (Mu et al., 2015). Interestingly, in the study of esophageal adenocarcinoma, *IGF2BP2* was found to be more highly expressed in metastatic lesions than in primary tumors (Barghash et al., 2016).

Through GSEA analysis, we found that the mechanism of *IGF2BP2* in HNSCC may also include the Notch signaling pathway, ERBB signaling pathway, catabolism, lipid metabolism, and amino acid metabolism. In conclusion, our study demonstrated that *IGF2BP2* is highly expressed in HNSCC tumor tissues and is associated with tumor progression and poor survival. This study provides new and promising insights for subsequent research to elucidate the molecular pathogenesis of HNSCC. Further mechanistic experiments and randomized clinical trials are required to identify the underlying mechanisms and clinical applications for HNSCC patients.

## Data Availability Statement

All datasets generated for this study are included in the article/supplementary material.

## Author Contributions

The project proposed by WC. ZL provided paraffin tissue sections and assisted in editing the manuscript. XD downloaded and mined the relevant data of TCGA and wrote the manuscript. QJ did immunohistochemistry and assisted in organizing data.

## Conflict of Interest

The authors declare that the research was conducted in the absence of any commercial or financial relationships that could be construed as a potential conflict of interest.

## Footnotes

1 https://portal.gdc.cancer.gov/repository

2 http://molpath.charite.de/cutoff/load.jsp
